# Interaction between microRNA and KRAS in Glioblastoma

**DOI:** 10.2174/0109298673343892241207034138

**Published:** 2025-01-03

**Authors:** Ozal Beylerli, Elmar Musaev, Gervith Reyes Soto, Carlos Castillo Rangel, Manuel De Jesus Encarnacion Ramirez, Tatiana Ilyasova

**Affiliations:** 1 Central Research Laboratory, Bashkir State Medical University, Ufa, Republic of Bashkortostan, 3 Lenin Street, 450008, Russia;; 2 Department of Oncology, Sechenov First Moscow State Medical University, Moscow, Russia;; 3 Department of Head and Neck, Unidad de Neurociencias, Instituto Nacional de Cancerología, 14080, Mexico City, Mexico;; 4 Department of Neurosurgery, Servicio of the 1ro de Octubre Hospital of the Instituto de Seguridad y Servicios Sociales de los Trabajadores del Estado, Mexico City, 07760, Mexico;; 5 Department of Neurosurgery, Russian People’s Friendship University, 121359, Moscow, Russia;; 6 Department of Internal Diseases, Bashkir State Medical University, Ufa, Republic of Bashkortostan, 450008, Russia

**Keywords:** KRAS gene, GBM, miRNA, gliomagenesis, therapy, targets

## Abstract

Glioblastoma (GBM) characterized byits rapid progression and challenging prognosis, often featuring mutations in the Kirsten rat sarcoma virus (KRAS) gene, which is crucial for numerous cellular signaling mechanisms. Emerging research underscores a significant interaction between KRAS and microRNAs (miRNAs) in these cancers, with miRNAs playing key roles as both regulators and mediators within the KRAS signaling framework. The concept of oncogene-induced senescence (OIS) is explored as a protective mechanism against tumor development, examining how K-RAS signaling is meticulously adjusted to bypass senescence, thereby enhancing cell growth and survival. In this study, we identify certain miRNAs that directly impact KRAS through mRNA targeting or by influencing its downstream signaling cascades. In turn, pathways activated by KRAS can modify the levels of specific miRNAs, establishing a feedback loop that balances cell regulation and tumor progression. We propose a theoretical framework where these interactions are crucial for deciphering the molecular underpinnings of GBM, potentially paving the way for innovative treatment approaches that focus on the miRNA-KRAS connection.

## 1 INTRODUCTION

**Fig. (1):**
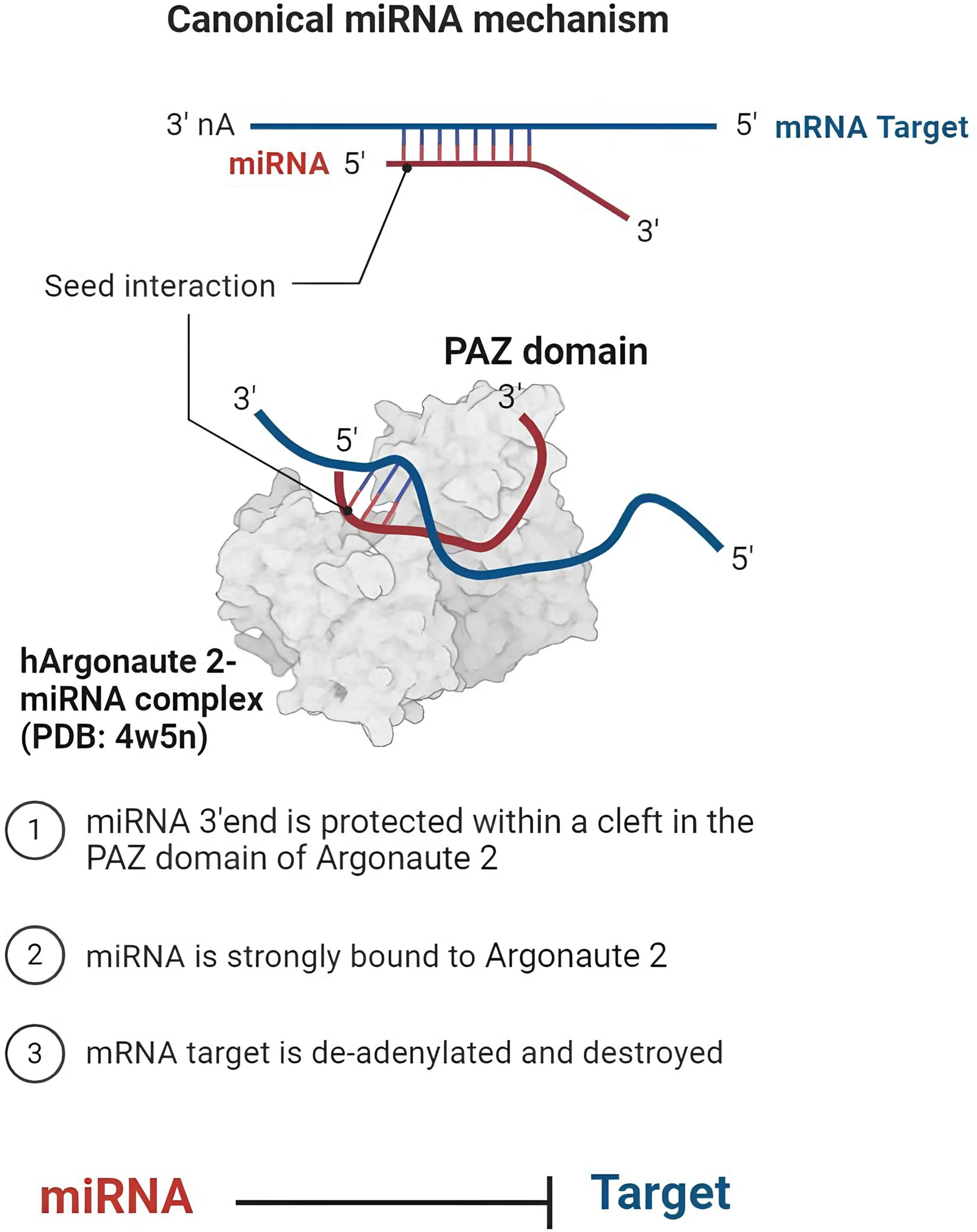
Canonical miRNA mechanism.

Glioblastoma (GBM) is recognized as the most malignant and prevalent form of brain tumor, characterized by its swift progression and profound resistance to existing therapeutic regimens [1]. This formidable disease underscores the critical need for breakthroughs in medical research and treatment strategies. The molecular intricacies of GBM reveal a complex interplay of genetic and epigenetic factors, among which the interaction between miRNAs and the Kirsten rat sarcoma virus (KRAS) gene plays a pivotal role. MicroRNAs (miRNAs), small non-coding RNA (ncRNA) molecules, are crucial in regulating gene expression at the post-transcriptional level and affect a myriad of cellular processes including cell proliferation, apoptosis, and migration (Fig. **1**) [2-6].

Innovative research in molecular biology has shed light on the significant impact of miRNAs in controlling oncogenic signaling pathways, with the KRAS protein serving as a key node in several critical pathways such as RAF/MEK/ERK and PI3K/AKT. These pathways are essential for maintaining tumor cell survival, promoting proliferation, and conferring resistance to therapy. Interestingly, while mutations in KRAS are uncommon in GBM compared to other cancers, the activity of KRAS can be significantly influenced by various miRNAs, affecting tumor behavior and the efficacy of treatment protocols.

This review delves into the nuanced interactions between miRNAs and KRAS within the specific context of GBM, aiming to highlight how these regulatory RNAs influence KRAS signaling. We will explore the mechanisms through which miRNAs impact KRAS activity and the downstream effects on GBM's pathophysiology. The discussion aims to uncover novel insights into how miRNA-based strategies could potentially temper the aggressive nature of GBM, paving the way for developing more effective therapeutic interventions. Through a comprehensive understanding of these molecular interactions, the goal is to enhance the prognosis and quality of life for patients afflicted with this aggressive disease, offering hope through potential new avenues for treatment.

## 2 PRINCIPLES OF KRAS SIGNALING AND MIRNA REGULATION

**Fig. (2):**
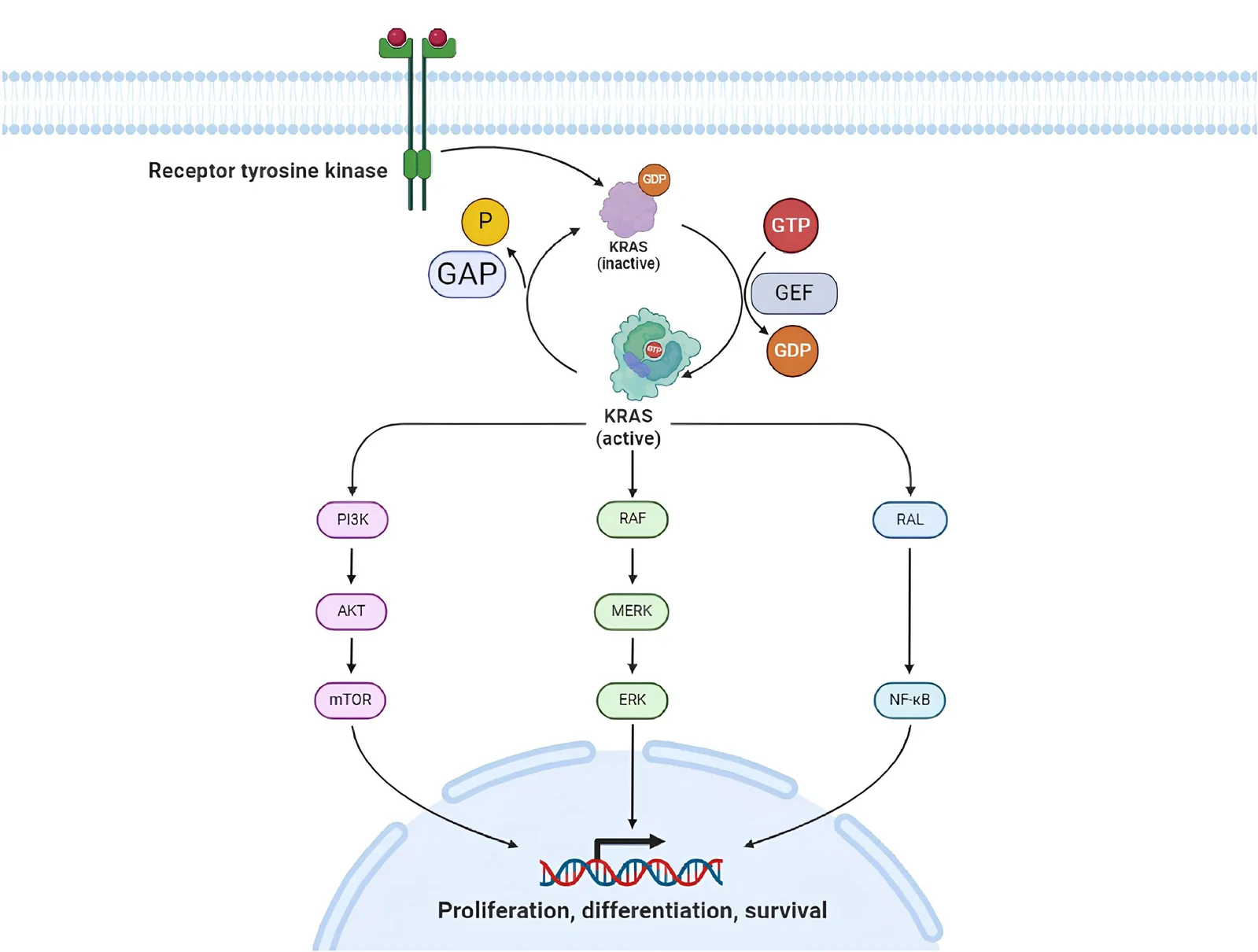
The regulation of KRAS activation and signal transduction.

KRAS, a key member of the membrane-associated monomeric GTPase family, acts as a fundamental molecular switch in cells, regulating responses to external signals, especially those from growth factors. Its activation hinges on guanine nucleotide exchange factors (GEFs), which facilitate the release of guanosine diphosphate (GDP) from KRAS, subsequently enabling guanosine triphosphate (GTP) binding. This GDP-GTP exchange is essential, as the attachment of GTP triggers KRAS to adopt an active conformation, allowing it to engage with multiple downstream signaling pathways that drive various cellular functions (Fig. **2**).

Once in its active state, KRAS engages with multiple critical signaling cascades, notably the RAF/MEK/ERK, PI3K/AKT, and RalGDS pathways. These pathways play essential roles in driving cellular responses related to proliferation, survival, and differentiation. KRAS's precise modulation of these cascades underscores its function as a crucial regulator of cellular homeostasis, ensuring that cells respond appropriately to various environmental signals and maintain stability in their physiological functions.

The activity of KRAS is terminated through the hydrolysis of GTP to GDP, a reaction sped up by GTPase-activating proteins (GAPs). GAPs increase KRAS’s inherent GTPase activity, facilitating its rapid switch from an active, GTP-bound state to an inactive, GDP-bound form. This quick deactivation process is vital for the fine-tuning of cellular signaling, allowing KRAS to exercise tight control over downstream pathways. The temporally precise modulation of these pathways is crucial for preventing overactive signaling, which could lead to uncontrolled cell growth or other detrimental effects [7].

However, oncogenic mutations in KRAS often disrupt this balance by either enhancing the exchange of GDP for GTP or impairing the GTPase activity, leading to a persistent, active state dominated by the GTP-bound form of KRAS. This constitutive activation drives prolonged signaling through its downstream pathways, which can result in excessive cell proliferation, survival, and tumorigenesis. Such mutations pose a significant challenge in cancer biology, as they make KRAS a primary driver of malignant progression [8]. A critical question from the standpoint of drug development is whether glioblastoma cells continue to rely on KRAS expression and activity for their growth and survival. This dependency would determine the potential efficacy of KRAS-targeted therapies in glioblastoma, emphasizing the importance of further research into KRAS's role and influence in these tumors.

Such mutations may maintain activation of downstream signaling pathways beyond their normal duration, promoting uncontrolled cell proliferation and survival, key processes in tumorigenesis. Recent discoveries in KRAS signaling have highlighted the role of miRNAs in its regulation [9, 10]. miRNAs can influence KRAS activity both directly by targeting KRAS mRNA and indirectly by affecting the expression and function of GEFs, GAPs, and various downstream effectors. This posttranscriptional regulation by miRNAs adds layer of complexity to the control of KRAS signaling, illustrating a complex network in which miRNAs serve as important modulators of KRAS function and associated pathways. Understanding the principles of KRAS signaling is fundamental to understanding its role in cellular physiology and the pathology of diseases in which KRAS is disrupted. The complex regulation of KRAS by both protein factors and miRNAs highlights its importance as a therapeutic target in KRAS mutant cancers, where normal regulatory mechanisms are often disrupted.

## 3 MIRNA-MEDIATED REGULATION OF KRAS IN CANCER

**Fig. (3):**
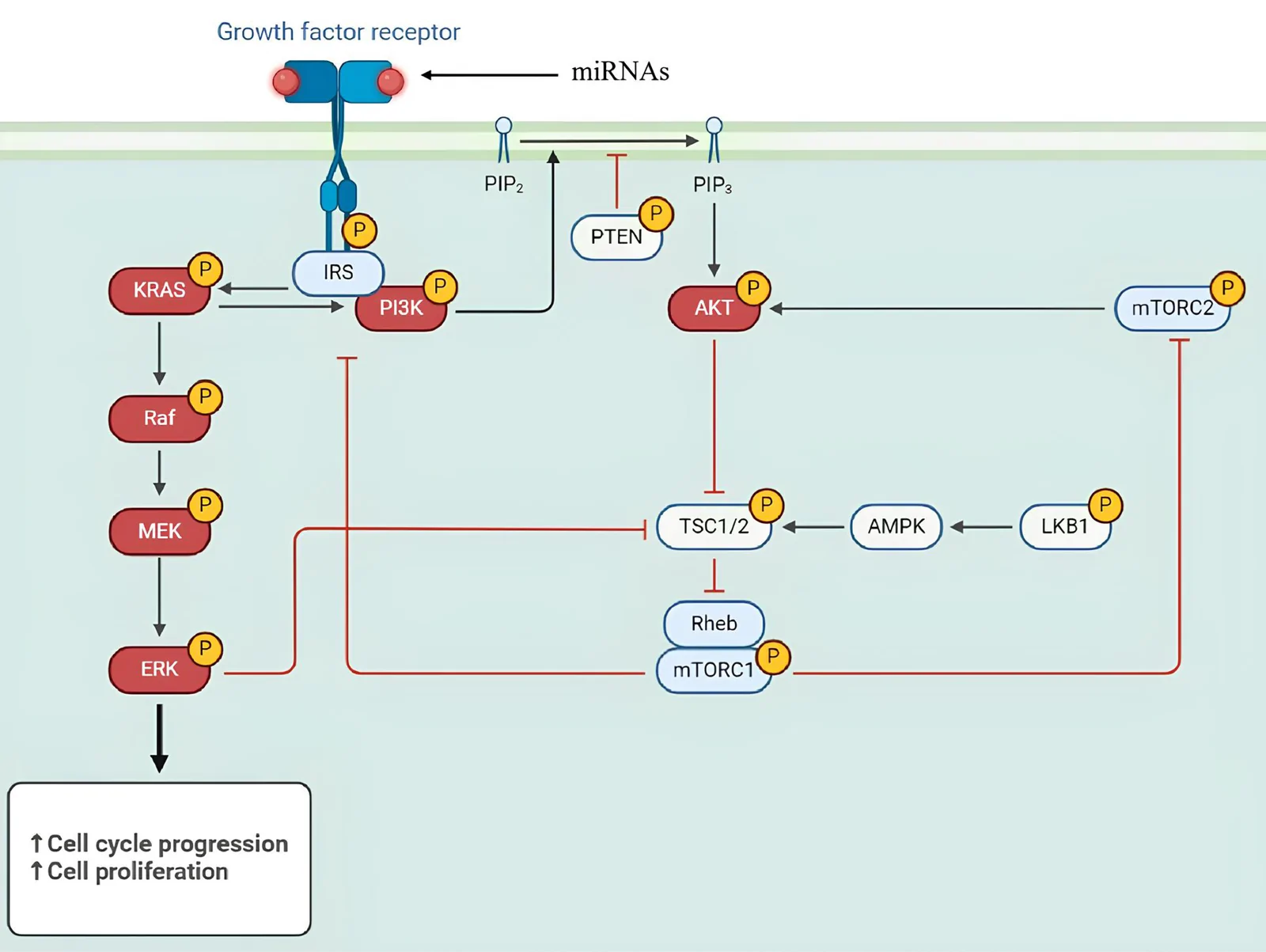
The oncogene role of miRNAs as and KRAS in RAF/MEK/ERK and PI3K/AKT pathways. For instance, miR-30c and miR-21 in non-small-cell lung cancer (NSCLC) or miR-31 in pancreatic and colorectal cancer.

MiRNAs that target and regulate KRAS have been identified as essential tumor suppressors, significantly influencing cancer progression. The seminal study by Johnson *et al.* first identified the let-7 family as tumor-suppressive miRNAs capable of directly targeting and regulating KRAS, establishing let-7's pivotal role in inhibiting oncogenic signaling pathways through KRAS regulation [11]. This discovery laid the groundwork for understanding miRNAs as potent suppressors in cancer biology. Subsequent research has highlighted additional tumor-suppressive miRNAs, such as miR-96, miR-30c, and miR-181a, which also regulate KRAS in various cancers. These miRNAs serve as inhibitors of KRAS-driven pathways, contributing to the suppression of cell proliferation and survival, which are crucial in limiting cancer progression [12, 13]. By targeting KRAS, these miRNAs interfere with the pathways responsible for unchecked tumor growth, thereby affirming their value as tumor suppressors. More recently, Gastaldi *et al.* utilized large-scale profiling technologies, including small RNA sequencing, to examine miRNA expression in cutaneous squamous cell carcinomas (cSCCs). This study identified the miR-193b/365a cluster as one of the most notably down-regulated miRNA clusters during murine skin tumor development. The tumor-suppressive function of this miRNA cluster was validated in both mouse and human epidermis, where it was shown to influence critical processes such as cellular proliferation, migration, and clonogenic potential. Functional assays further confirmed that miR-193b/365a negatively regulates KRAS, with an inverse relationship observed between these miRNAs and KRAS protein levels. Moreover, the knockdown of KRAS in squamous carcinoma cells replicated the suppressive effects seen with miR-193b/365a expression, further supporting its tumor-suppressive role through KRAS targeting [14]. While multiple studies report deregulation of various miRNAs in cancer, only a few have been thoroughly characterized. For instance, Liao *et al.* explored miR-30b, which is down-regulated in colorectal cancer (CRC), in a cohort of 91 CRC patients. The study showed that lower levels of miR-30b correlated with poor disease progression and reduced patient survival [15]. In experimental models, overexpression of miR-30b in CRC cell lines and xenograft mice resulted in tumor suppression, primarily through G1 cell-cycle arrest and induction of apoptosis. The tumor-suppressive effects of miR-30b are mediated by its regulation of multiple genes, including KRAS, demonstrating its significant role in inhibiting tumor growth and confirming its therapeutic potential in CRC (Fig. **3**) [15].

**Fig. (4):**
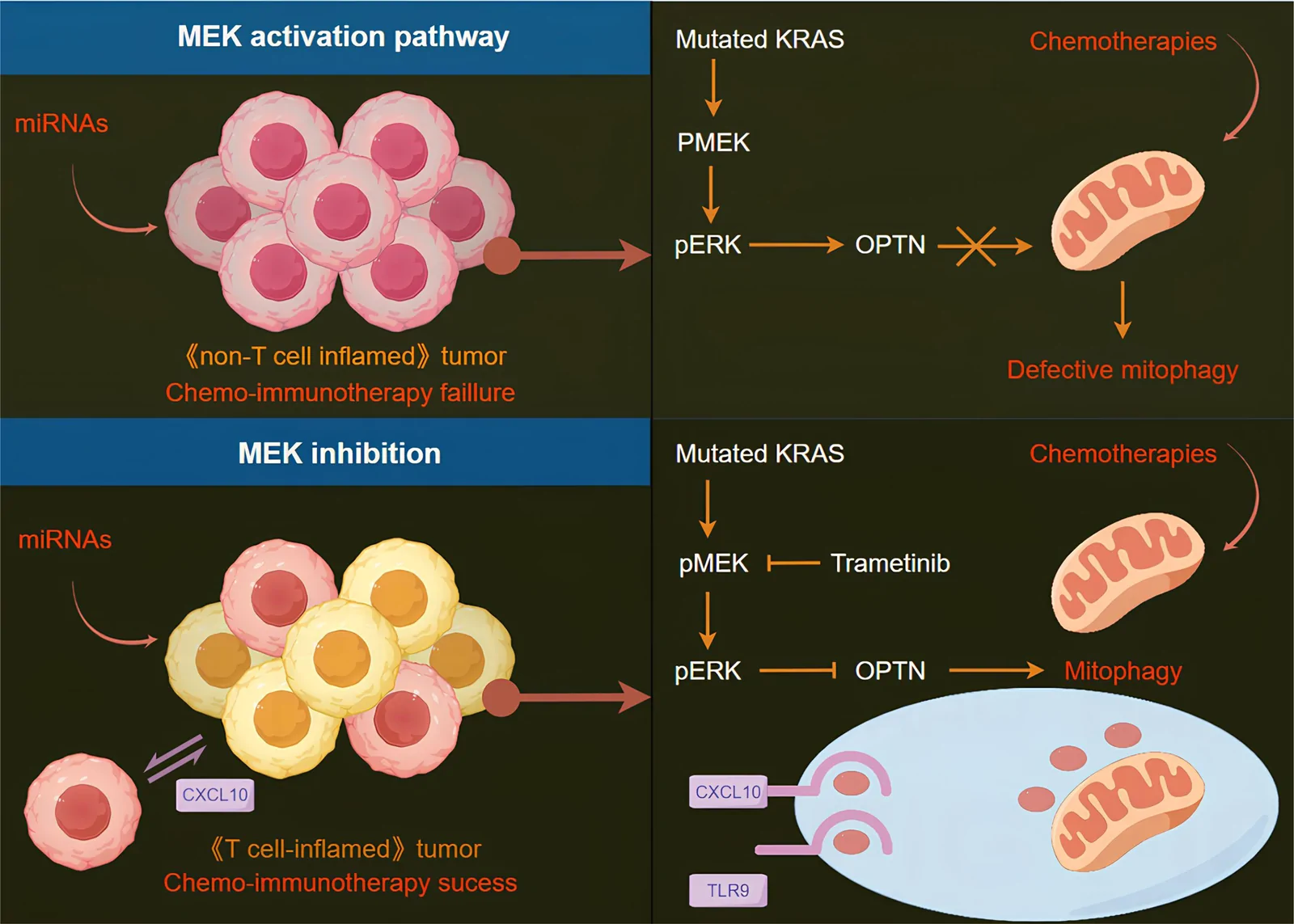
The role of miRNAs and KRAS mutation in MEK pathway. For instance, tumor-suppressive effects of miR-335 colon cancer and miR-98 in retinoblastoma; oncogene role of miR-139 in NSCLC.

In addition to miRNA dysregulation, sequence variations within the 3′ untranslated region (UTR) of target mRNAs can substantially influence gene regulation by modifying miRNA binding effectiveness. Analysis of the 3′ UTR region of KRAS across several non-small cell lung cancer (NSCLC) cases led researchers to identify rs61764370, often called the KRAS-variant, as the first single nucleotide polymorphism (SNP) within a complementary site for the let-7 miRNA. This specific SNP has garnered attention as a potential biomarker indicating susceptibility to NSCLC (Fig. **4**) [16].

Moreover, beyond NSCLC, the KRAS-variant has demonstrated potential as a risk biomarker for a range of cancers, as well as for conditions like endometriosis [17, 18]. Additionally, it has been explored as a predictor of response to certain therapeutic agents [19]. However, further studies are required to establish its reliability across diverse patient populations. Several studies have reported an absence of association between the KRAS-variant and cancer risk [20, 21] or drug response in particular cases [20-22]. For instance, large-scale clinical trials examining stage 3 colon cancer found no significant link between this variant and cancer stage, and another study on endometrial cancer failed to find an association, likely due to sample size limitations [23, 24]. In addition to the KRAS-variant, researchers continue to investigate other SNPs within the 3′ UTR of KRAS that may serve as cancer risk biomarkers. While less extensively validated, another SNP, rs712, has shown potential as a biomarker for cancers such as oral squamous cell carcinoma, gastric cancer, colorectal cancer, and papillary thyroid cancer [25, 26]. However, a recent study by Kim *et al.* did not identify any new NSCLC-associated variants, possibly due to the small sample size used [27]. Despite these findings, research by Kim *et al.*, along with Sabarinathan *et al.*, suggests that certain SNPs might disrupt miRNA-mediated regulation of KRAS by altering miRNA binding sites or modifying the RNA's secondary structure [28]. Such disruptions can interfere with KRAS regulation, affecting its expression levels and highlighting the role of 3′ UTR SNPs in influencing cancer susceptibility and progression by disrupting miRNA targeting.

## 4 MIRNA AND KRAS INTERACTION IN GBM

Recent studies underscore the vital role of miR-134 in glioblastoma multiforme (GBM), the most prevalent and lethal form of brain cancer. Research has shown that decreased levels of miR-134 are inversely associated with receptor tyrosine kinase (RTK) activity, particularly MET, EGFR, and PDGFR. Restoration of miR-134 expression has been found to enhance the efficacy of RTK inhibitors, underscoring the therapeutic potential of modulating this miRNA in GBM treatment [29]. This study sheds light on the mechanisms by which RTKs influence miR-134 and its effects on GBM *via* pathways like MAPK, facilitated by the transcription factor KLF4. Functionally, miR-134 acts as a tumor suppressor by inhibiting growth, survival, and stemness in cancer cells, directly targeting oncogenes such as KRAS and STAT5B, making it a key target for future GBM therapies [29-31].

Additional investigations reveal that dysregulated miR-134 plays a critical role in glioma pathogenesis, especially through its interaction with the ERK signaling pathway, a major controller of cell proliferation and tumor growth [30]. Experimental manipulation in glioma cell line U251, through increased miR-134 expression, demonstrated reduced cellular proliferation and invasion and identified KRAS as a direct miR-134 target. miR-134 binds to the 3′-UTR of KRAS mRNA, thereby reducing KRAS expression and subsequent ERK pathway activation, leading to decreased tumor progression. Further validation showed that overexpressing KRAS without the 3′-UTR could partially counteract miR-134’s suppressive effects, highlighting the specificity and importance of miR-134’s interaction with KRAS in inhibiting glioma progression [30].

Research has also explored the epigenetic regulation of miR-134 in gliomas, revealing its substantial downregulation in high-grade tumors. Treatment with 5-azacytidine (5-AZA), a demethylating agent, restored miR-134 expression, which correlated with reduced tumor cell proliferation, invasion, and progression in both *in vitro* and mouse models. This study positions miR-134 as a significant biomarker for glioma and a viable therapeutic target due to its regulatory effects on KRAS, as well as its role in modulating both the ERK and AKT signaling pathways in glioma development [31].

Within cancer genetics, RAS oncogenes, including KRAS, are recognized as prominent regulators across numerous cancer types. Although RAS mutations are uncommon in brain tumors, the RAS/MAPK pathway frequently remains activated through other genomic abnormalities (*e.g.*, mutations in EGFR, PDGFR, NF1), serving as a central mechanism of gliomagenesis. Importantly, let-7, a well-established tumor-suppressive miRNA, regulates RAS family genes such as KRAS, significantly reducing cell proliferation and migration in GBM models (U251, U87) both *in vitro* and *in vivo*. This miRNA specifically targets tumor cells while sparing normal astrocytes, emphasizing its potential for selective and targeted treatment of GBM [32-34]. Studies on let-7a, a variant of the let-7 family, have further confirmed its tumor-suppressive effects in glioma. Low levels of let-7a expression are associated with higher tumor grades and poorer prognosis in GBM patients. Functionally, let-7a induces cell cycle arrest, promotes apoptosis, and inhibits migration through KRAS regulation, with these effects shown to be independent of PTEN status, a key tumor suppressor often implicated in glioma pathology. This independence highlights let-7a’s specific pathway regulation within glioma cells [34].

In other research, Li *et al.* examined miR-126, noting its marked downregulation in GBM tissues and correlation with higher tumor grades. miR-126 interacts directly with the 3′-UTR of KRAS, suppressing the ERK pathway and thereby inhibiting the proliferation and invasion of glioma cells. The study suggests that enhanced miR-126 expression may serve as a diagnostic biomarker and a therapeutic target, with the potential to impede glioma progression by modulating KRAS and other key oncogenic pathways [35].

**Fig. (5):**
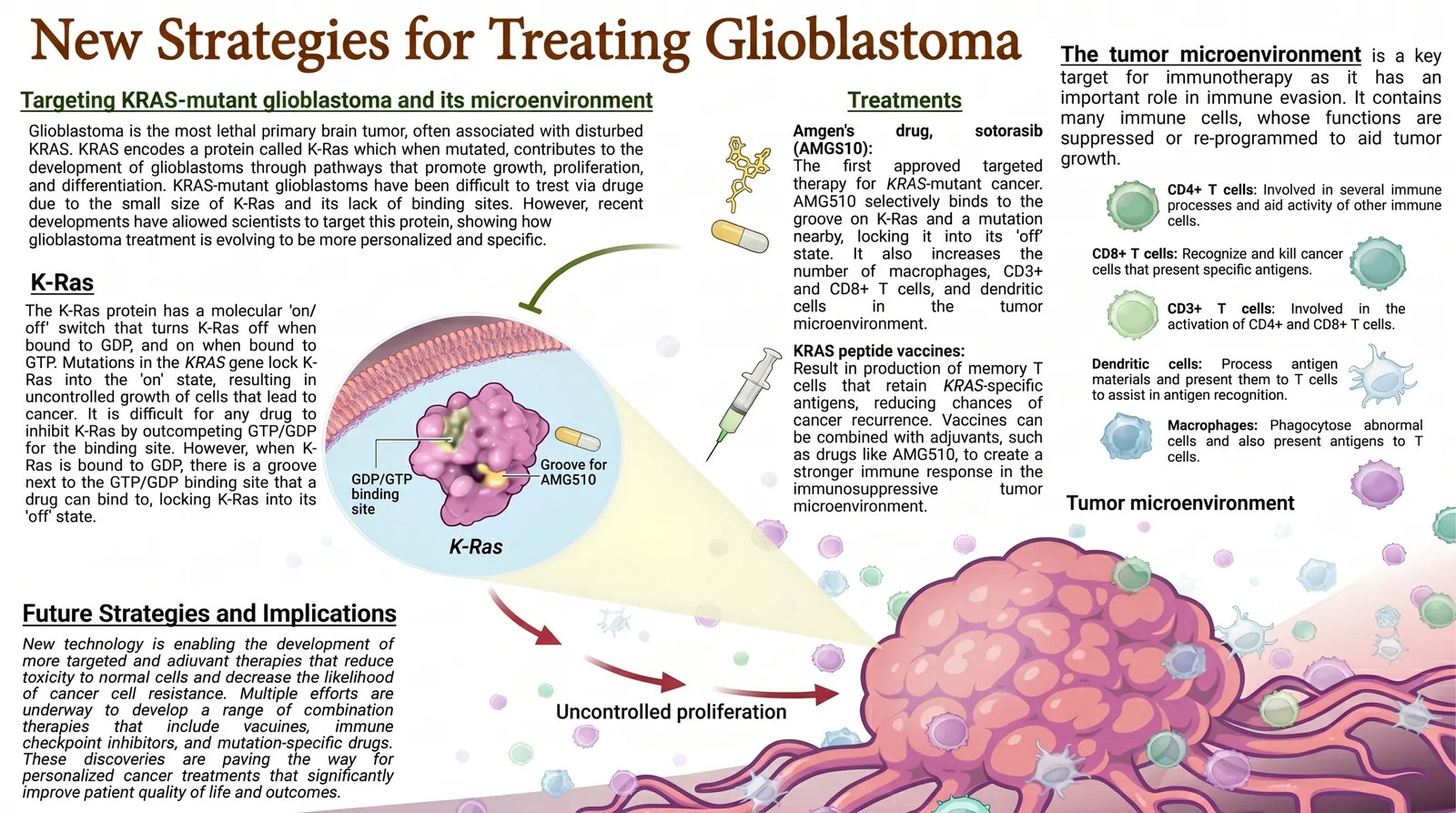
New strategies for treating GBM.

In GBM treatment, radiation therapy is critical yet often hindered by therapeutic resistance and tumor recurrence. Research has identified a cellular response mechanism whereby ionizing radiation activates the KRAS/ERK pathway, leading to increased CD44 expression, a surface protein that supports stemness and invasion. This process, modulated by suppressed miRNAs like miR-202 and miR-185, exacerbates resistance to therapy. Targeting the KRAS/ERK/CD44 axis could mitigate these effects, improving radiotherapy effectiveness and addressing the challenges of tumor recurrence (Fig. **5**) [36].

 Another study by Li *et al.* focused on miR-199a, identifying it as a tumor suppressor with reduced expression linked to poor prognosis in GBM. miR-199a directly inhibits KRAS, suppressing both the AKT and ERK pathways and increasing sensitivity to the chemotherapy agent temozolomide (TMZ). This miR-199a/KRAS axis highlights a promising approach to address chemoresistance, offering a novel avenue for therapeutic intervention in resistant GBM [37].

Finally, research by Wang *et al.* demonstrated that miR-181d inhibits glioma proliferation by simultaneously targeting KRAS and Bcl-2. Reduced miR-181d levels in gliomas result in enhanced cell survival through the activation of PI3K/AKT and MAPK/ERK pathways. By modulating these key survival pathways, miR-181d emerges as a potential therapeutic agent for GBM, highlighting the significant promise of miRNA-based strategies in combating this aggressive cancer (Table **1**) [38].

## CONCLUSION

This review underscores the pivotal role of miRNAs in modulating KRAS signaling pathways, which are instrumental in GBM pathogenesis, specifically influencing cell proliferation, survival, and resistance to therapies. Key miRNAs, such as miR-134, let-7, miR-126, miR-199a, and miR-181d, have been identified as significant regulators of KRAS, directly impacting downstream pathways like RAF/MEK/ERK and PI3K/AKT that drive GBM’s aggressive phenotype. By targeting these miRNAs, it may be possible to modulate KRAS activity, providing a new avenue for therapeutic intervention aimed at reducing tumor progression and enhancing sensitivity to existing treatments [9, 39].

The therapeutic potential of miRNA-based approaches lies in their ability to offer a multifaceted intervention: not only can they suppress oncogenic signaling, but they may also counteract GBM’s notorious resistance to therapies such as radiation and chemotherapy. For example, reintroducing or mimicking tumor-suppressive miRNAs could inhibit KRAS-driven pathways that sustain GBM growth and resilience, while reducing adverse effects on normal cells. Additionally, the unique profiles of miRNAs in GBM offer a promising pathway for their use as biomarkers, enabling more precise patient stratification and potentially guiding personalized treatment strategies.

Looking forward, research should prioritize the clinical translation of miRNA-based therapies, focusing on optimizing delivery mechanisms, assessing long-term efficacy, and minimizing off-target effects. Expanding our understanding of the miRNA-KRAS regulatory network and its interactions with other oncogenic pathways in GBM will be essential to advancing these miRNA-based strategies. Ultimately, harnessing miRNAs to modulate KRAS activity could revolutionize GBM treatment, offering hope for improved prognosis and tailored therapies in this challenging and aggressive cancer. 

## AUTHORS’ CONTRIBUTIONS

The authors confirm their contributions to the paper as follows: conceptualization, writing-original draft preparation, writing-review and editing: O.B., E.M.; validation, investigation, resources, and visualization: O.B., T.I.; acquisition, analysis, and interpretation of the data: G.R.S., C.C.R.; supervision and project administration: E.M.; funding acquisition: O.B., M.J.E.R. All authors have read and agreed to the published version of the manuscript.

## Figures and Tables

**Table 1:** Comprehensive overview of key miRNAs, target genes, pathways, findings, and therapeutic implications in GBM.

miRNA	Targeted Genes/ Pathways	Experimental Models	Key Findings	Therapeutic Implications	References
miR-134	KRAS, STAT5B (MAPK, RTK signaling)	GBM tissues and cell lines; RTKs and miR-134 interactions	miR-134 acts as a tumor suppressor by modulating KRAS and STAT5B	Enhances RTK inhibitor efficacy, promising target for GBM treatment	[29-31]
let-7	KRAS, pan-RAS, NRAS (RAS/MAPK)	GBM cell lines (U251, U87); *in vitro* and *in vivo*	let-7 limits proliferation, tumor size *via* KRAS regulation	Selective cancer-targeting potential, minimal effect on normal cells	[32-34]
miR-126	KRAS (ERK pathway)	GBM tissues; tumor grade correlation assays	miR-126 downregulation correlates with high tumor grade, inhibits ERK pathway	Potential biomarker for high- grade gliomas and target for therapy	[35, 36]
miR-199a	KRAS (AKT, ERK pathways)	GBM cell lines; xenograft model for chemo-sensitivity	miR-199a targets KRAS to enhance TMZ chemo-sensitivity in GBM	Strategy to overcome chemoresistance in GBM	[37]
miR-181d	KRAS, Bcl-2 (PI3K/AKT, MAPK/ERK)	Glioma tissues; human cell lines; mouse xenografts	miR-181d reduces proliferation by dual targeting of KRAS and Bcl-2	Novel potential for GBM therapy *via* dual-targeted approach	[38]

## LIST OF ABBREVIATIONS

GBM: Glioblastoma
KRAS: Kirsten Rat Sarcoma Virus
OIS: Oncogene-Induced Senescence
miRNAs: MicroRNAs
ncRNA: non-coding RNA
GEFs: Guanine Nucleotide Exchange Factors
GDP: Guanosine Diphosphate
GTP: Enabling Guanosine Triphosphate
